# Injection of Varicose Veins

**Published:** 1890-07-05

**Authors:** 


### SURGERY.

### INJECTION OF VARICOSE VEINS.

The obliteration of varicose veins in the lower extremities by means of ligature or even radical surgical procedures, has for a long time been preferred to attempts at attaining the same end by injections, as being free from the danger of embolism, &c., attendant on the coagulation of the blood. But Dr. Mollière ("Lyon Médicale," 1890, No. 13) recommends the latter, which is less formidable to the patient if not so to the surgeon, but substitutes for the old Liq. Ferri. Perchlor, the scarcely less powerful, though non-irritant styptic Iodo-Tannin; his formula being Iodine 1 gram, Acid Tannic 9 grams, distilled water 200 grams. He directs the patient to walk briskly for half an hour or an hour before the operation so as to cause the veins to dilate to their utmost capacity; he then applies a firm bandage below the point where the incision or puncture is to be made and injects the varix with a few drops of the solution by means of a Pravaz syringe with a double (*sic*) canula. The puncture is then sealed with iodoform collodion, and the bandage being left on the limb the patient is strictly confined to bed for a fortnight, when a hard cord only remains, which in course of time disappears by absorption. Admitting that in iodotannin we have the least irritating of styptics we should ourselves prefer to ligature the enlarged vein above and below the point of injection as a further precaution against the transport of portions of the coagulum into the vena cava and right side of the heart.

